# A longer wood growing season does not lead to higher carbon sequestration

**DOI:** 10.1038/s41598-023-31336-x

**Published:** 2023-03-11

**Authors:** Roberto Silvestro, Qiao Zeng, Valentina Buttò, Jean-Daniel Sylvain, Guillaume Drolet, Maurizio Mencuccini, Nelson Thiffault, Shaoxiong Yuan, Sergio Rossi

**Affiliations:** 1 Laboratoire sur les écosystemes terrestres boreaux, Département des Sciences Fondamentales, Université du Québec à Chicoutimi, 555 boulevard de l’Université, Chicoutimi, QC G7H2B1 Canada; 2 Guangdong Open Laboratory of Geospatial Information Technology and Application, Guangzhou Institute of Geography, Guangdong Academy of Sciences, Guangzhou, 510070 People’s Republic of China; 3 Forest Research Institute, Université du Québec en Abitibi-Témiscamingue, Rouyn-Noranda, QC Canada; 4 Direction de la recherche forestiere Ministère des Forêts, de la Faune et des Parcs, Québec, QC G1P3W8 Canada; 5 Centre de Recerca Ecològica i Aplicacions Forestals (CREAF), 08193 Bellaterra, Barcelona Spain; 6 Institució Catalana de Recerca i Estudis Avançats (ICREA), Passeig de Lluis Companys 23, 08010 Barcelona, Spain; 7 Canadian Wood Fibre Centre, Canadian Forest Service, Natural Resources Canada, 1055, du P.E.P.S., Sainte-Foy Stn., P.O. Box 10380, Quebec, QC G1V 4C7 Canada; 8 Centre for Forest Research, Faculty of Forestry, Geography and Geomatics, Université Laval, 2405 rue de la Terrasse, Quebec, QC G1V 0A6 Canada

**Keywords:** Ecophysiology, Forest ecology

## Abstract

A reliable assessment of forest carbon sequestration depends on our understanding of wood ecophysiology. Within a forest, trees exhibit different timings and rates of growth during wood formation. However, their relationships with wood anatomical traits remain partially unresolved. This study evaluated the intra-annual individual variability in growth traits in balsam fir [*Abies balsamea* (L.) Mill.]. We collected wood microcores weekly from April to October 2018 from 27 individuals in Quebec (Canada) and prepared anatomical sections to assess wood formation dynamics and their relationships with the anatomical traits of the wood cells. Xylem developed in a time window ranging from 44 to 118 days, producing between 8 and 79 cells. Trees with larger cell production experienced a longer growing season, with an earlier onset and later ending of wood formation. On average, each additional xylem cell lengthened the growing season by 1 day. Earlywood production explained 95% of the variability in xylem production. More productive individuals generated a higher proportion of earlywood and cells with larger sizes. Trees with a longer growing season produced more cells but not more biomass in the wood. Lengthening the growing season driven by climate change may not lead to enhanced carbon sequestration from wood production.

## Introduction

Wood is one of the largest terrestrial carbon pools on Earth and a natural and renewable resource. Over the last decades, there has been growing interest in applied wood science. Firstly, as a raw material and all its derived products, wood is of crucial importance in the global bioeconomy and world trade^1^. Several research teams are exploring the possibility that wood can substitute fossil fuels and non-biomass materials^2,3^, especially in the construction industry^3,4^. In addition, wood growth counteracts global warming by carbon sequestration^5^. However, despite the economic and ecological roles that wood plays, our understanding of its formation and the factors influencing the resulting tree-ring structure remains incomplete.

Phenology is a crucial driver of tree fitness and species distribution^6^. Outside the tropics, meristems follow alternating periods of activity and dormancy according to the annual cycle of the seasons. Plants use environmental triggers (e.g., temperature, water, solar radiation) to synchronize the timings of growth and reproduction with favorable conditions during the year. The timing of phenological events is thus calibrated to ensure the optimal conditions to complete all stages of the annual life cycle while minimizing the risk of damage^7^.

Wood formation dynamics are key drivers of the variation in wood features across the growth ring^8^. The ratio between cell diameter and cell wall thickness discriminates between earlywood and latewood cells, which result from different temporal dynamics of their cellular trait formation. Earlywood cells undergo a longer duration of enlargement than latewood cells, forming bigger cells with lower cell wall thickness^8^. Latewood cells are subjected to a longer duration of secondary wall deposition, leading to smaller cells with thicker cell walls. These changes in the temporal dynamics of earlywood-latewood formation lead to different sensitivities of wood formation to endogenous and environmental factors during the growing season. In conifers, earlywood is more dependent on soil water content to sustain a greater cell extension, and latewood depends more often on temperature and sugar availability. The availability of this last resource increases when shoot elongation ends^9–11^.

Variability within xylem anatomical traits, i.e., tracheid diameter and wall thickness, reflects structural and physiological trade-offs at the base of tree functioning and performance^12^. Indeed, wider tracheids are more efficient in transporting water but possibly more susceptible to cavitation^13^. In contrast, narrower thick-walled tracheids afford most of the mechanical support and hydraulic safety but are less conductive^13^. Anatomical traits and tracheid morphology also drive many fundamental wood properties and play a crucial role in determining the quality of forest-derived products^14^. Among all wood traits, density has a major impact on wood quality and the value of resulting wood products and composites^15^. Wood density is the measure of the total amount of cell wall material available per volume unit. Tissue properties such as earlywood tracheid diameter, the proportion of latewood and, specifically, cell-wall thickness are major factors explaining variation in the intra-ring wood density^14,16^. Therefore, geometric models based on cell anatomical features have been used to calculate the so-called morphometric density^17^, which represents an acceptable simplification, even if only partially explaining the density variation along the growth ring^16^.

Wood formation dynamics and the duration of differentiation stages influence cell traits^8^. However, wood phenology can vary significantly among individuals^18,19^. For this reason, it is worth exploring the consequences of this variability on the relationship between the phenology of wood formation and anatomical characteristics and the resulting effects on wood density. This study evaluates the variability among individuals in xylem developmental dynamics and the related anatomical traits during the growing season 2018 in a balsam fir [*Abies balsamea* (L.) Mill.] stand.

The study aims to explore the relationships and trade-offs among wood formation temporal dynamics, annual productivity, and wood anatomical traits. Based on an intra-annual scale, we will first quantify the inter-individual variability in wood phenology^19^ and then test whether a longer duration of the growing season corresponds to a greater cell production^19,20^. Subsequently, we test three alternative hypotheses related to the anatomical traits of xylem. A longer growing season leads to a greater annual tracheid production in which: (1) Earlywood and latewood productions increase proportionally, no differences in morphometric density can be highlighted; (2) A longer growing season increases carbon assimilation in wood^21^, latewood production augments and thus increases carbon sequestration and morphometric density; or (3) An earlier onset of the growing season does not result in extra carbon sequestration from wood production^22^, earlywood production increases, resulting in a lower morphometric wood density.

## Results

### Wood formation dynamics

The first enlarging cells of earlywood, corresponding to the onset of wood formation, were observed from mid-May to late June between the day of the year (DOY) 136 and 178. More than 58% of the trees started cell differentiation between DOY 149 and 157 (Figure S1). On average, cell wall thickening and lignification started 13 days after the onset of cell enlargement, between early June and early July (DOY 158–184), with 71% of the trees beginning between DOY 163 and 170 (Figure S1). The first mature cells of earlywood were detected 10 days after the onset of cell wall thickening and lignification, between late June and early July (DOY 171–191), with 79% of the trees starting between DOY 173 and 182 (Figure S1).

The first enlarging cells of latewood were observed 53 days after the onset of earlywood differentiation, between DOY 190 and 243, corresponding to the period between early July and late August (Figure S1). The onset of cell wall thickening and lignification of latewood occurred 33 days later than the onset of cell enlargement of latewood. About 63% of the sampled trees started cell wall thickening and lignification of latewood between mid and late July (DOY 194–207) (Figure S1). The first mature latewood cell was observed 14 days after the onset of cell-wall thickening and lignification of latewood. The first mature latewood cell was observed between late July and early September (DOY 200–251), occurring in late August (DOY 233–242) in more than 40% of the trees (Figure S1).

**Figure 1:**
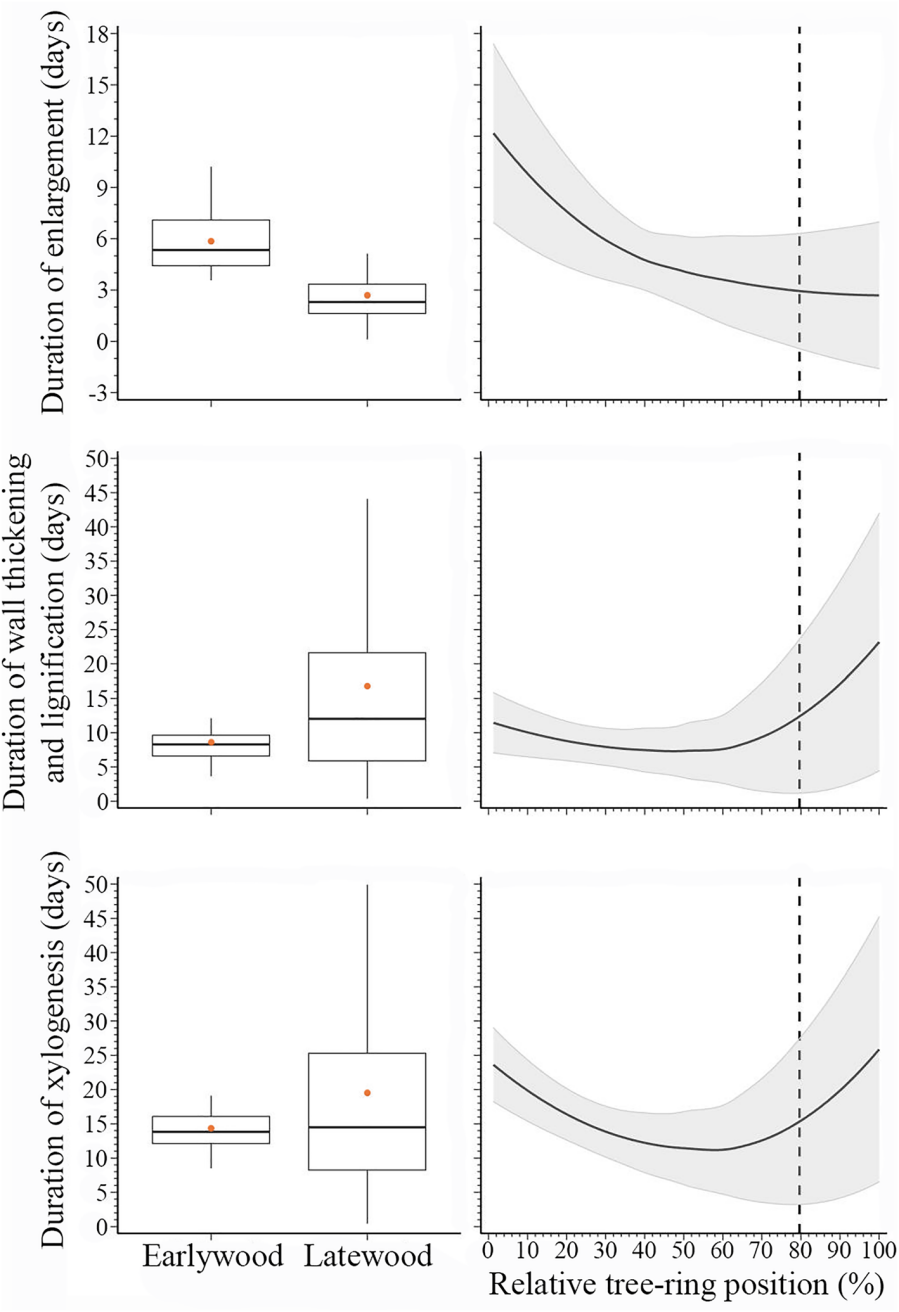
Duration of xylem formation (cell enlargement and secondary cell-wall deposition) across the growth ring in 27 balsam firs at the Montmorency Forest (QC, Canada). Boxplots represent upper and lower quartiles; whiskers achieve the 10th and 90th percentiles; the median and mean values are drawn as horizontal black lines and black dots, respectively. The trends result from loess function (span 0.9), with the grey background representing the standard deviation. The dotted line represents the boundary between earlywood and latewood.

On average, 93% of the studentized residuals of the duration of wood formation obtained by the fitting of the loess function ranged between -1.96 and 1.96, demonstrating that the model adequately represented the average pattern of the duration of wood formation (Figure S2). On average, the duration of cell enlargement of a tracheid lasted 5 days. The longest duration (12 days) of cell enlargement was observed for the first xylem cells, while the development of enlarging cells lasted only 3 days at the end of the growth ring (Fig. 1). On average, earlywood cell enlargement duration (6 days) was higher than that of latewood (3 days). Overall, cell wall thickening and lignification duration showed a higher variation in latewood, lasting 9 and 17 days in earlywood and latewood, respectively. At the first percentile of the growth ring, xylem cells required 11 days to complete cell-wall thickening and gradually decreased to 7 days at 50% of the growth ring, then increased to 23 days at its end. Compared to earlywood, latewood showed higher variability in the duration of xylogenesis. On average, earlywood and latewood cells required 15 and 20 days to complete xylogenesis, respectively. According to loess function, the duration of xylogenesis was 24 days for the first earlywood cell and decreased to 11 days at 60% of the growth ring, while it increased to 26 days for the last latewood cell (Fig. 1).

### Cell production and anatomical traits

**Figure 2:**
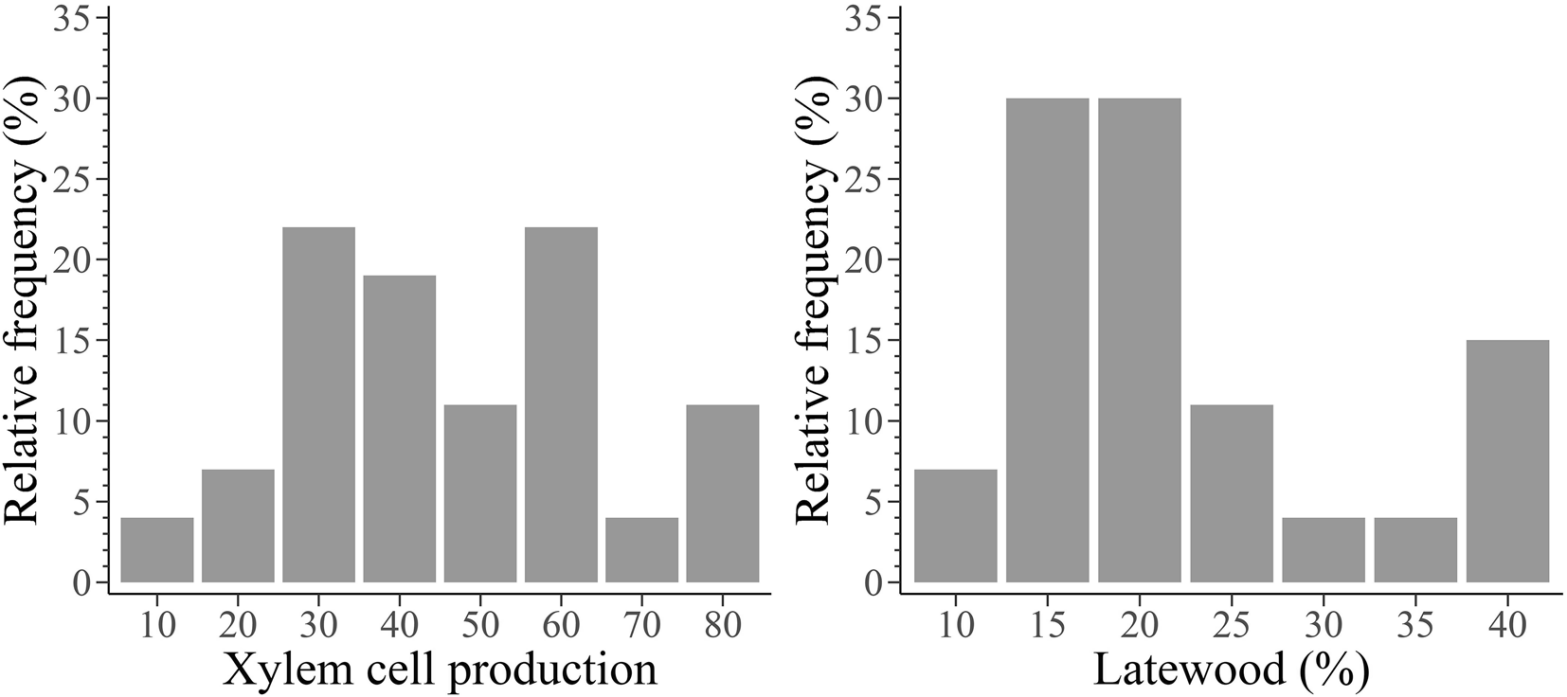
Xylem cell production and latewood cell percentage in 27 balsam firs at the Montmorency Forest (QC, Canada).

Xylem cell production varied highly among trees, resulting in a range of between 8 and 79 cells observed at the end of the growing season (Fig. 2). Latewood cells varied from 6 to 40%. 60% of trees showing 12–24% of latewood (Fig. 2).

**Figure 3:**
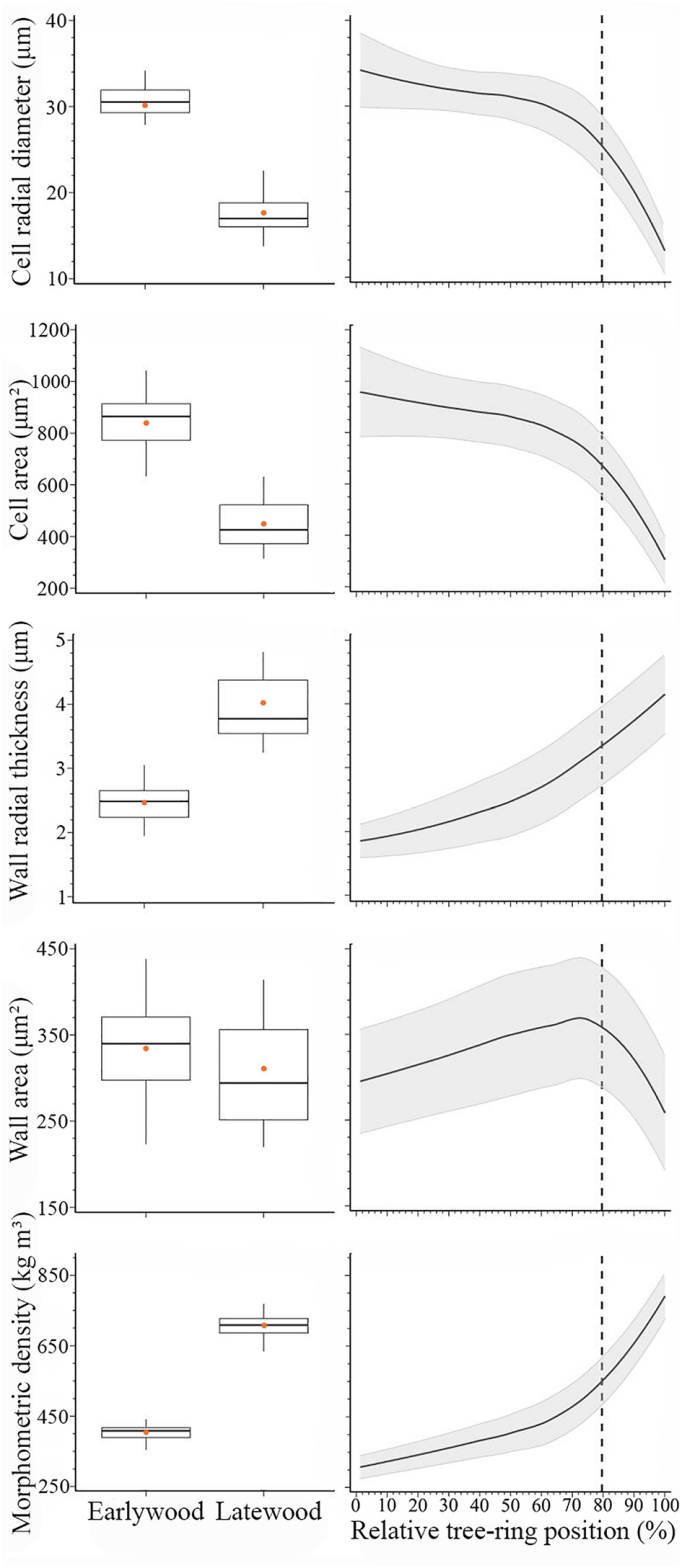
Wood anatomical traits and morphometric density across the growth ring in 27 balsam firs at the Montmorency Forest (QC, Canada). Boxplots represent upper and lower quartiles; whiskers achieve the 10th and 90th percentiles; the median and mean values are drawn as horizontal black lines and black dots, respectively. The trends result from loess function (span 0.9), with the grey background representing the standard deviation. The dotted line represents the boundary between earlywood and latewood.

The studentised residuals of the wood anatomical traits resulting from the fitting of the loess function exhibited no trend, with random distribution around zero (Figure S3). On average, 94% of the studentised residuals showed an absolute value of ± 1.96, indicating that the model appropriately represented the trends of wood anatomical traits (Figure S3). In general, cell diameter ranged from 13.1 to 34.2 μm, with a mean of 28.8 μm. Cell radial diameter decreased gradually during earlywood production (average value of 31.2 μm) but sharply declined during latewood formation (average value of 19.7 μm). The largest standard deviations (± 4.3 μm) were observed at the beginning of wood formation, decreasing towards the end of the growth ring (± 2.5 μm) (Fig. 3). The variation of cell area had a similar pattern to cell radial diameter (Fig. 3). The largest cell area (958.0 μm^2^) was observed from the first earlywood cells, decreasing to 305.3 μm^2^ at the end of the growth ring. Wall radial thickness gradually increased from 1.9 to 4.2 μm during the tree-ring formation, with an average of 2.7 μm across the growth ring. Cell wall area slowly increased from 295.3 μm^2^ at the first percentile of the growth ring, culminated at 369.1 μm^2^ at 73% of the growth ring, and reduced during latewood production. The standard deviation of the cell wall area was relatively constant (± 67.4 μm^2^ on average) across the growth ring (Fig. 3). The values of morphometric density ranged from 307.9 to 791.0 kg m^−3^, with an average of 449.5 kg m^−3^ (Fig. 3). The morphometric density was lower across the earlywood and higher in latewood, which matched the pattern of cell wall thickness.

### Wood formation dynamics, production and cell traits

**Figure 4:**
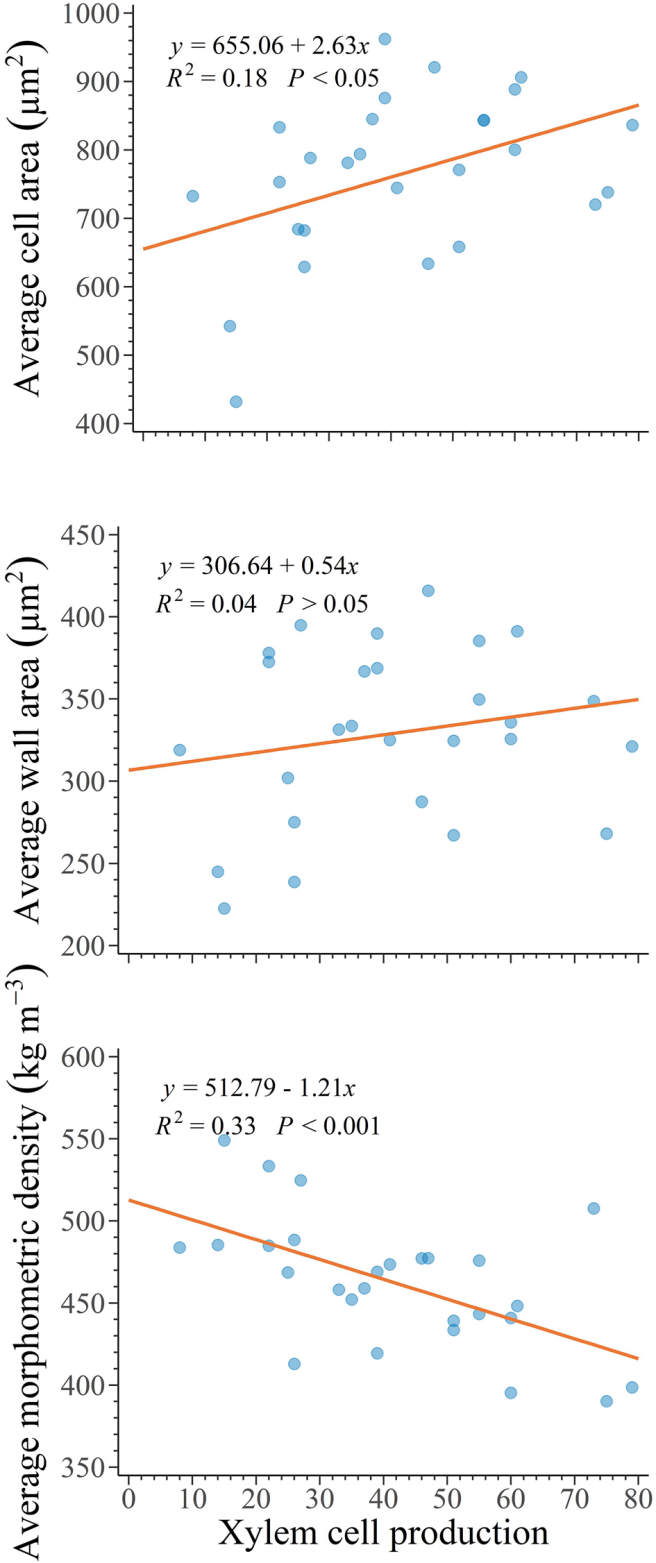
Relationships between cell production and anatomical traits in 27 balsam firs at the Montmorency forest (QC, Canada).

Cell production exhibited a positive linear relationship with cell-wall area (*p* < 0.05, R^2^ = 0.18, Fig. 4). The linear relationship between cell production and cell wall area was also positive but not statistically significant (*p* > 0.05, R^2^ = 0.04, Fig. 4). We observed a negative relationship between cell production and average morphometric density (*p* < 0.001, R^2^ = 0.33, Fig. 4), indicating that the morphometric density progressively declines as cell production increases.

**Figure 5:**
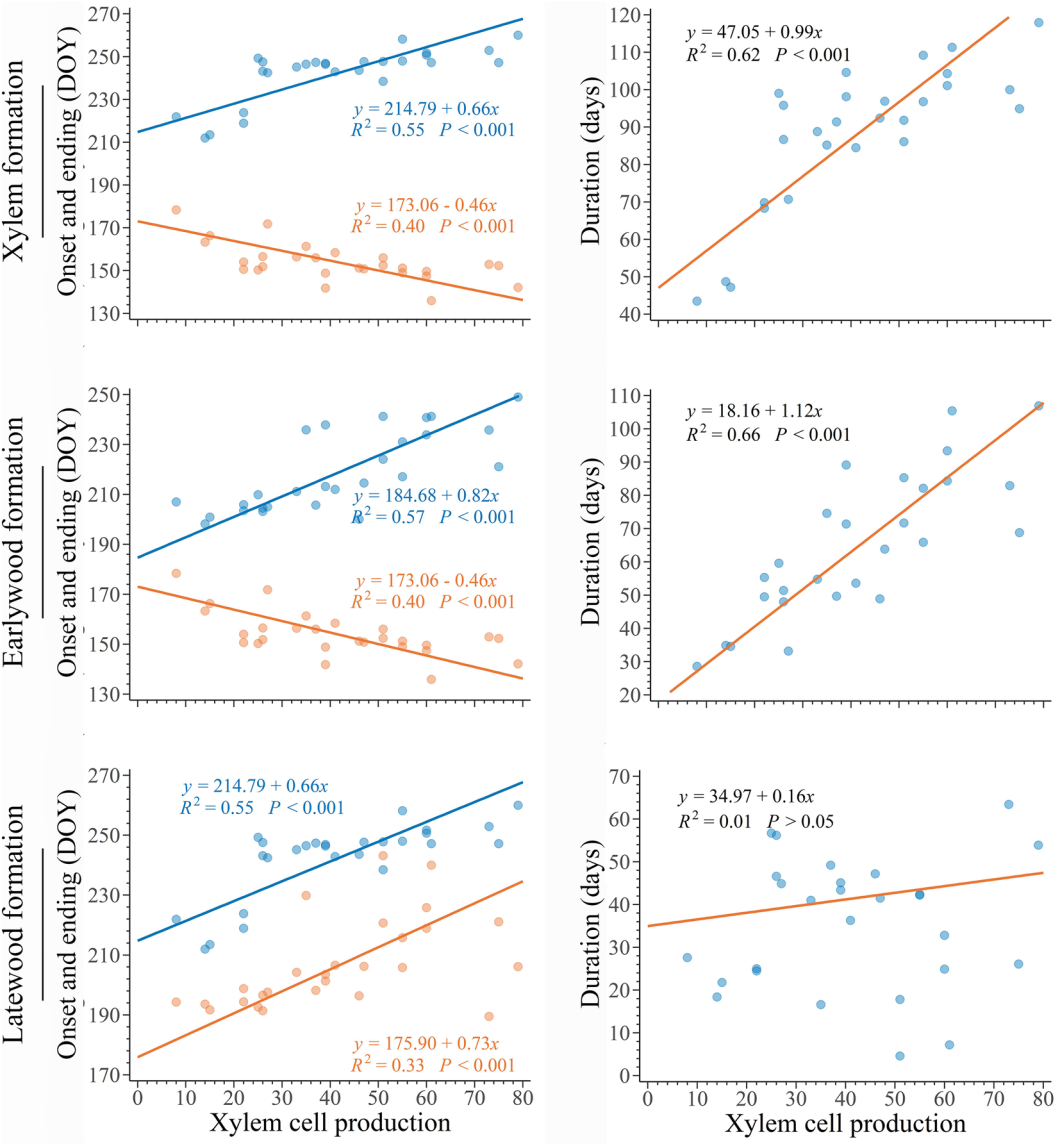
Standardized major axis (SMA) regressions among timings of onset (orange dots) and ending (blue dots) of xylem phenology, earlywood and latewood phenology as a function of the total number of cells in the growth ring at the end of the growing season in 27 balsam firs at Montmorency forest (QC, Canada).

**Figure 6:**
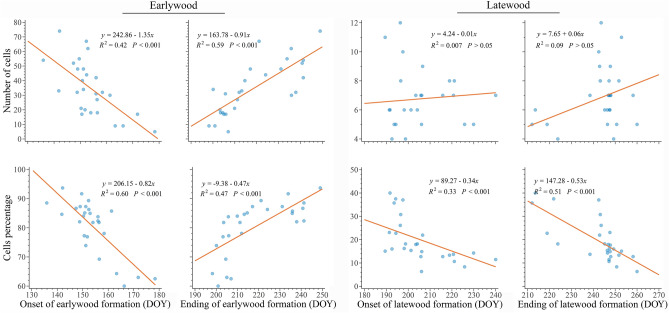
Relationships between the amount of earlywood and latewood (expressed as cell number and percentage of total cell production) and earlywood and latewood phenology in 27 balsam firs at Montmorency forest (QC, Canada).

The relationships between cell production and the onset, ending, and duration of xylem formation were highly significant (*p* < 0.001, R^2^ of 0.40, 0.55 and 0.62, respectively, Fig. 5). These relationships indicate that an earlier growth resumption and later growth cessation, which correspond to a longer growing season, lead to greater annual cell production. This trend was maintained when the relationship between the onset, ending, and duration of earlywood formation was examined (*p* < 0.001, R^2^ of 0.40, 0.55 and 0.66, respectively, Fig. 5). Considering the relationship between latewood formation and annual cell production, both a later onset and later ending were positively correlated with a greater xylem cell production (*p* < 0.001, R^2^ of 0.33 and 0.55, respectively, Fig. 5), which led to a non-significant relationship between the length of latewood formation and annual production of xylem cells (*p* > 0.05, Fig. 5). According to the previous results, we observed that a longer growing season, with earlier onset and later ending, increased both the number of earlywood cells and their percentage out of the total annual production (*p* < 0.001, with R^2^ ranging from 0.42 to 0.60, Fig. 6). On the contrary, the relationships (*p* > 0.05) between the number of latewood cells and the timings of onset and ending of their production were not significant (R^2^ of 0.007 and 0.09, respectively, Fig. 6). The percentage of latewood of the total cell production was correlated with both the onset and the ending of its formation (*p* < 0.001, R^2^ of 0.33 and 0.51, respectively, Fig. 6). That means that the longer latewood production is delayed during the growing season the more earlywood is produced.

**Figure 7:**
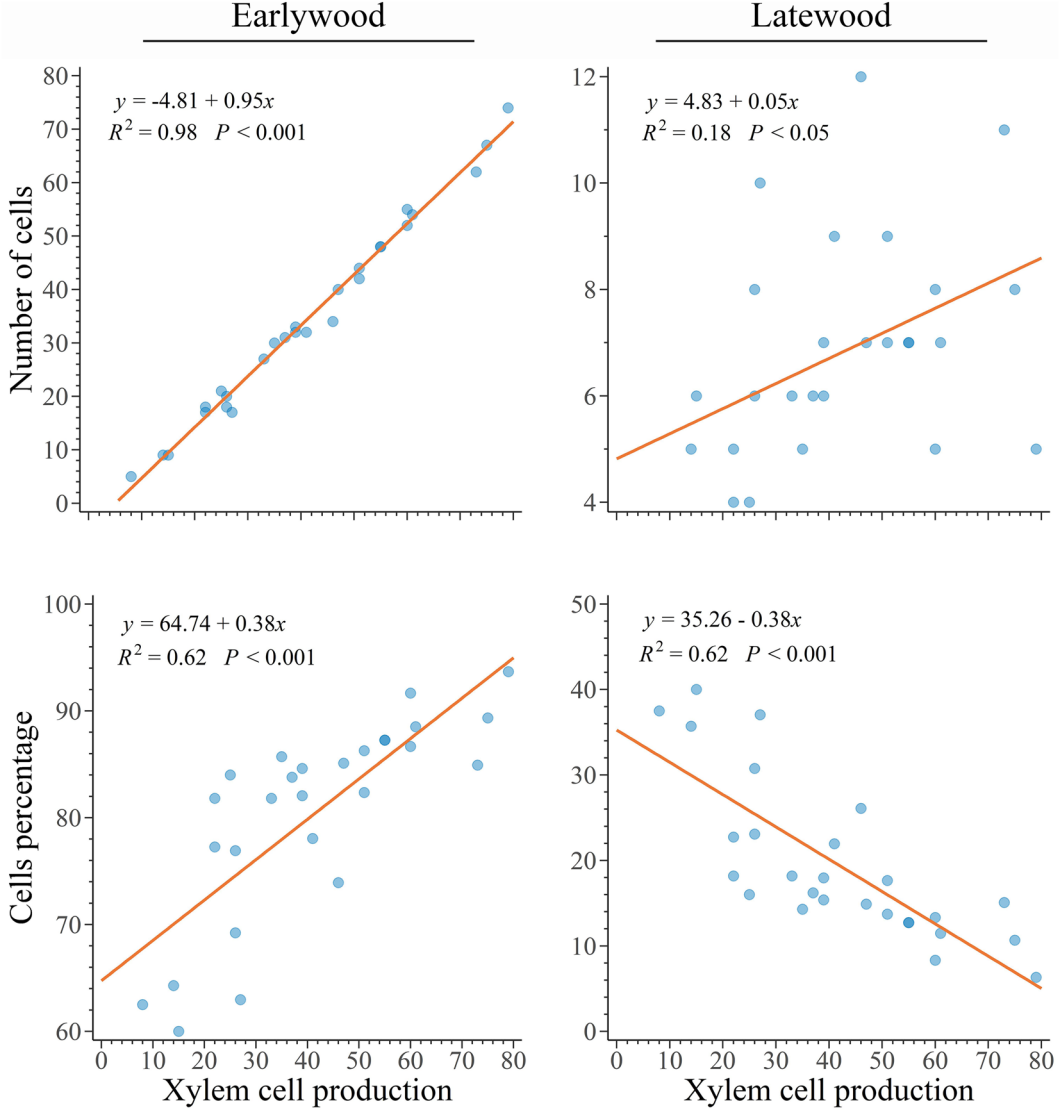
Relationships between the amount of earlywood and latewood (expressed as cell number and percentage of total cell production) and annual cell production in 27 balsam firs at Montmorency forest (QC, Canada).

The linear relationship between number of earlywood and latewood cells and total xylem cell production was significant and positive (*p* < 0.001 and 0.05, R^2^ of 0.98 and 0.18, Fig. 7), demonstrating that the production of both earlywood and latewood cells increases with greater cell production. However, the rate of increase differs between earlywood and latewood, with earlywood cells representing 95% of all tracheids produced. This became evident when observing the ratio between earlywood and latewood cells. Indeed, while we observed an increase in the percentage of earlywood (*p* < 0.001, R^2^ = 0.62, Fig. 7), latewood percentage decreased with annual cell production (*p* < 0.001, R^2^ = 0.62, Fig. 7). Therefore, a greater cell production corresponds to a longer duration of the growing season and a greater production in earlywood compared to latewood.

## Discussion

This study investigated the intra-annual dynamics of wood formation in 27 balsam firs at the Montmorency Forest, Quebec, Canada. Trees with a higher cell production experienced a longer duration of the growing season, represented by an earlier onset and a later ending of wood formation. More productive individuals also generated a higher proportion of earlywood and, at the same time, of cells with larger dimensions, resulting in a lower morphometric wood density than individuals with lower cell production. These results support the initial hypothesis that, at the intra-annual scale, individuals experiencing an earlier resumption of xylem growth increase earlywood production and decrease morphometric wood density.

### More is less: phenology, productivity and cell traits

According to our results, the number of cells produced at the end of the growing season is related to the duration of xylogenesis. Trees starting xylem differentiation earlier and ending later, thus experiencing a longer growing season, have a higher cell production. Moreover, the number of earlywood and latewood cells increases in trees with a longer growing season. However, the production does not increase proportionally. Still, we observed a longer duration and greater production of earlywood cells and, consequently, a reduction in latewood percentage in the tree-ring in trees with a longer growing season. The disproportional augmentation in earlywood production over latewood finally results in a higher proportion of larger cell sizes and, thus, a lower morphometric density.

This relationship between wood phenology, annual cell productivity and the related cell traits may be explained by physiological processes affecting primary and secondary growth. At the beginning of the growing season, xylem development depends on the influx of auxin from bud primordia^23^, where this hormone is primarily produced, to then be conveyed into polar auxin via phloem transport^24^. Consequently, activation of the apical meristems is essential for the onset of wood phenology, which could explain their synchronism^11^.

Primary and secondary growth processes depend on one another, are synchronous, and unquestionably competitive^11,25^. Therefore, primary growth can potentially play a role in contributing to defining the ratio between earlywood and latewood. Once the synchronous resumption of primary and secondary meristem activity begins in the early spring, sink competition for carbon allocation is unavoidable^9^. Specifically, when shoots and needles in the canopy are actively growing, the wood formation process has a lower allocation priority of the recently assimilated C. As for secondary growth, a large variability is found in the timings and duration of primary growth phenological phases^7,26,27^. Given the synchronism between primary and secondary meristematic activity^11^ and the influence of primary growth on sugar availability for xylogenesis^9,28^, the timing of bud break and duration of shoot extension can likely influence the timing of secondary growth resumption but also the related cell traits and the ratio between earlywood and latewood. The result of this close interaction is that an anticipated bud break and longer duration of shoot extension could drive a greater earlywood production, given that this type of xylem cells also relies on C assimilated from the previous growing seasons^29–31^. At the end of summer, once the shoot extension is completed, the priority in C allocation changes, and latewood formation can finally profit from an increased and continuous carbon supply^9,11^.

These close interactions between primary and secondary growth can explain why the difference in the rates of increase between earlywood and latewood correlated to a longer growing season, as observed in the present study. Therefore, if our hypothesis is correct, anatomical patterns in wood formation could be directly determined by the C allocation patterns during the growing season.

### Variability at the onset and end of the growing season

The variability among trees in the duration of wood formation increased from the onset to ending of the growing season. The source of this variability along the tree-ring lay in the larger variability in the duration of cell wall deposition during latewood formation. However, this variability in the secondary cell wall deposition duration was independent of the individual variability in anatomical traits along the growth ring.

A complex relationship exists between cell traits and the duration of their differentiation phases^9,12^. In our study, the duration of cell enlargement is longer during earlywood than latewood development. This result agrees with other studies performed on several conifer species in which larger earlywood cells were coupled with a longer duration of cell enlargement^8,17,32^. Despite the difference in the duration of cell enlargement between early and latewood formation, the variability among trees remained constant during the growing season.

At the end of the growing season, the duration of cell wall thickening drives the timing of latewood formation^8,9,25^. According to our data, the duration of cell wall thickening is characterised by a large variability among individuals. Our results agree with previous studies^19^, in which the end of the growing season was marked by a larger among-tree variability than the onset. The difference in variability between the onset and the ending of wood phenology is likely related to a different contribution of the endogenous and environmental factors driving these phenological phases. Indeed, while environmental and overall weather conditions drive growth resumption^33,34^, internal factors, such as the timing and duration of resumption of primary growth, as well as the amount of cell production and water and sugar availability, are likely involved in defining the duration and the ending of wood formation^35–37^.

Balsam fir is known for the low quality of its wood, especially regarding its mechanical properties^38^. However, the same xylem anatomical features of balsam fir explain its great capacity for physiological adjustments (e.g. high light-use efficiency, a low respiration rate, and a long seasonal period of active photosynthesis)^39–41^. The highlighted relationships between phenological timings, annual productivity and the resulting anatomical traits of the growth ring may also be found in other species. However, the degree of variability among individuals might differ, for example in species with a slower growth habit and a more pronounced conservative behavior in wood formation.

### Growing season length and carbon sequestration

Our study analyzed patterns at intra-annual scale by focusing on processes occurring at a daily resolution during one growing season. However, some considerations can arise for a picture at larger scale. Our results confirm the relationships between the reduction in wood density and enhanced growth in volume of wood observed in the last century in Central Europe^42^. Over the last decade, the scientific literature provided evidence of a global acceleration of forest growth dynamics due to climate change^42–46^. It has been demonstrated that an earlier onset of the growing season induced by climate change does not result in enhanced carbon sequestration from wood production in temperate deciduous trees of North America^22^. Our results at the intra-annual scale confirm what was observed by Dow et al.^22^, and specifically that an increase in xylem cell production or related traits such as tree-ring width or volume of wood cannot be straightforwardly converted into sequestrated carbon, neither should we look at it as a biomass harvest potential. New insights into the relationships between wood functional traits can deliver crucial knowledge to upscale carbon sequestration from tissue-to-individual scale. However, assessment at intra-annual scale lacks the resolution to estimate the effect of weather on wood formation and how growth ring width relates to wood mass. In this context, the combination of tree ring analysis, quantitative wood anatomy, and the assessment of wood formation has the potential to quantify how climatic variations affect wood functional traits and the amount of carbon annually sequestered in the tree stem. Enhancing our accuracy in linking climatic features to carbon sequestration is a crucial step for making reliable ecological predictions in a changing climate that will unavoidably impact the forestry industry and the global market related to wood production.

## Conclusions

Our results show the high variability in wood phenology among trees of the same stand. A longer growing season corresponds to greater annual cell production. This interaction finally results in less dense wood due to a larger size of cells and lower proportion of latewood. Our study provides an eco-physiological explanation that reflects the interaction between the endogenous and environmental factors driving xylem phenology and wood production. We assume that temperature and precipitation play a key role in defining the timing of wood formation, while the pattern in C allocation during the growing season is involved in determining the wood anatomical traits. This interaction among multiple factors became even more complex when considering the weight of each factor and the period during the growing season.

These results about the relationships and interactions between phenological timings and the dynamics of cell trait development illustrate that our understanding of wood formation remains incomplete. Accordingly, a clear understanding of all the factors involved in the variability in wood phenology and production among trees still need further investigations.

## Materials and methods

### Study area

We conducted our study in the balsam fir–white birch bioclimatic domain at the Montmorency Forest (47° 16′ 20″ N, 71° 08′ 20″ W), Quebec, Canada. The study area has a typical continental climate with cool and humid summers and cold and long winters. The annual temperature is 0.5 °C during 1981–2010. July is the warmest month with a mean temperature of 14.6 °C. January is the coldest, with a mean temperature of -15.9 °C. Annual precipitation is 1583 mm, of which a third falls in the form of snow.

The study area covers 218 ha and was submitted to a clear cut during 1993–1994. The dominant species is balsam fir, presenting pure and mixed stands with a current age ranging between 25 and 30 years. The soil type is classified as Ferro-Humic Podzol and Humic Podzol^47^, covered by a 4.6 MOR humus layer.

### Xylem phenology

We selected 27 dominant balsam firs from permanent plots with healthy crowns and upright stems to assess wood formation from April to October 2018. The sampled trees were of the same age but different in height and diameter at breast height (DBH; 1.3 m). Microcores were collected weekly from each sampled tree using a Trephor^48^. To avoid the development of resin ducts, samples were collected 10 cm apart^49^.

The samples were dehydrated through successive immersion in ethanol and D-limonene, then embedded in paraffin^48^. Transverse sections of wood tissue 8 μm in thickness were cut with a rotary microtome and stained with cresyl violet acetate (0.16% in water)^50^. We discriminated between developing and mature tracheids under visible and polarised light at magnifications of × 400– × 500. The number of cambial, enlarging, cell wall thickening and lignifying and mature cells was counted along three radial rows. Specifically, cambial and enlarging cells remained dark under polarised light, indicating only primary cell walls. During the cell wall thickening and lignification phase, cells glistened under polarized light. Cell maturation was reached when they were completely blue^50^.

### Xylem cell anatomy

Two additional microcores per tree were collected at the end of summer 2018. We prepared the samples according to the abovementioned experimental protocol. Wood sections were stained in safranin (1% water) and stored on micro slides with a PermountTM mounting medium. We collected digital images of wood transversal sections with a camera fixed on an optical microscope at magnifications of × 20. We measured the lumen radial diameter, lumen tangential diameter, wall radial thickness, and lumen area for all the transversal sections using WinCELL™ (Regent Instruments, Canada).

Since cell size increases in the radial rather than the tangential direction during the enlarging phase, we used cell radial diameter to show the variation of the cell enlarging phase^17^. We calculated cell radial diameter as the sum of the lumen radial diameter and the two wall radial thicknesses. As wall tangential thickness was estimated to be 1.2 times wall radial thickness, we defined cell tangential diameter as the sum of lumen tangential diameter and 2 times wall tangential thickness (or 2.4 times wall radial thickness)^17^. As the shape of a cell in the transversal section was approximate to a rectangle, we computed cell area as the product of cell radial diameter and tangential diameter, and wall area was defined as the difference between cell and lumen area.

According to Mork’s criterion, latewood cells were defined as the tracheids showing a wall radial thickness four times larger than the lumen radial diameter^51^. We determined the percentage of latewood cells as the ratio of latewood cells to the total number of xylem cells at the end of the growing season^51^. The morphometric density was calculated as a function of the wall cross-sectional area, cell radial diameter and cell tangential diameter according to Cuny et al*.*^17^.

### Statistical analyses

Based on the raw data of the number of cells counted at each sampling date, generalised additive models (GAMs) were performed for each sampled tree to evaluate the annual cell production and both the timings and duration of each xylogenesis phenological phase^17^ and to generate the tracheidograms following Buttò et al*.*^8^. Accordingly, the duration of cell enlargement was defined as the difference between the onset of cell wall thickening and lignification and onset of cell enlargement. The duration of the cell wall thickening and lignification phase was defined as the difference between the date of mature cell and the onset of cell wall thickening and lignification, and the duration of xylogenesis was calculated as the period between the first mature cell and first enlarging cell^8^.

For each individual, we obtained the tracheidograms of cell diameter, cell area, wall thickness, wall area and morphometric density according to the percentile position of cells across the growth ring^8^. For each tree, we applied GAMs to fit the variations of these wood anatomical traits along with the relative tree-ring positions^8^. Loess function was performed to obtain the general patterns of the duration of wood formation and wood anatomical traits across the growth ring. We validated the reliability of fitting by performing the distribution of studentized residuals. Linear regressions were used to assess the relationship between cell production, anatomical traits and timings of xylogenesis. We used standardised major axis (SMA) linear regressions to test the relationship between the timings of xylem formation and cell production^19^. We used SMA regression as we could not state which one of the variables was independent. Therefore, the aim was to test the relationship between the variables and estimate the line best describing the scatter. All statistics were performed in R 3.6.1^52^.

## Supplementary Information

Supplementary Information.

## Data Availability

Data associated with this paper are available in Borealis: https://doi.org/10.5683/SP3/OTADBZ
