# Supplementary material for: A longer wood growing season does not lead to higher carbon sequestration
Source: Sci Rep. 2023 Mar 11;13:4059. doi: 10.1038/s41598-023-31336-x (PMC10008533; doi:10.1038/s41598-023-31336-x)
Supplement: Supplementary file 1 — Supplementary Information. [file 41598_2023_31336_MOESM1_ESM.docx]

**Supplementary Materials**

**Title:**

A longer wood growing season does not lead to higher carbon sequestration

**Authors:**

Roberto Silvestro^1, *^, Qiao Zeng, Valentina Buttò, Jean-Daniel Sylvain, Guillaume Drolet, Maurizio Mencuccini, Nelson Thiffault, Shaoxiong Yuan, Sergio Rossi.

^1^ Laboratoire sur les écosystèmes terrestres boréaux, Département des Sciences Fondamentales, Université du Québec à Chicoutimi, 555 boulevard de l’Université, Chicoutimi (QC) G7H2B1, Canada.

* Corresponding author: roberto.silvestro1@uqac.ca (RS)





**Figure S1** Modelized timings of xylem phenology in earlywood and latewood of 27 balsam firs at the Montmorency Forest, QC, Canada.





**Figure S2** Studentised residuals vs duration of wood formation phases resulting from the fitting of loess function. The dotted lines represent the range between -1.96 and 1.96.





**Figure S3** Studentised residuals vs wood anatomical traits resulting from the fitting of loess function. The dotted lines represent the range between -1.96 and 1.96.
